# MALDI-TOF MS monitoring of PBMC activation status in sepsis

**DOI:** 10.1186/s12879-018-3266-7

**Published:** 2018-07-31

**Authors:** Aurélie Daumas, Julie Alingrin, Richard Ouedraogo, Patrick Villani, Marc Leone, Jean-Louis Mege

**Affiliations:** 1 Aix-Marseille Université, URMITE, IHU Méditerranée Infection, UMR CNR 7278, IRD 198, INSERM 1095, Marseille, France; 2 Service de Médecine Interne et Thérapeutique, Hôpital de la Timone, Assistance Publique-Hôpitaux de Marseille, Marseille, France; 3 Service d’Anesthésie et Réanimation polyvalente, Hôpital Nord, Assistance Publique-Hôpitaux de Marseille, Marseille, France

**Keywords:** Sepsis, Mass spectrometry, MALDI-TOF, Mononuclear cells, IFN-γ, Interleukin-10, CpG oligonucleotides

## Abstract

**Background:**

MALDI-TOF mass spectrometry (MS) on whole cells enables the detection of different cell types and cell activation. Here, we wondered whether this approach would be useful to investigate the host response in sepsis.

**Methods:**

Peripheral blood mononuclear cells (PBMCs) from patients with severe sepsis and healthy donors were analyzed with MALDI-TOF MS. PBMCs from healthy donors were also stimulated with lipopolysaccharide, peptidoglycan*,* CpG oligonucleotides, polyinosinic polycytidylic acid, and with heat-inactivated bacteria. Averaged spectra of PBMCs stimulated in vitro by different agonists were generated from the database using the Biotyper software and matching scores between each spectrum from patients and averaged spectra from the database were calculated.

**Results:**

We show that the MALDI-TOF MS signature of PBMCs from septic patients was specific, compared with healthy controls. As the fingerprints observed in patients may be related to PBMC activation, PBMCs from healthy controls were stimulated with cytokines, soluble Pathogen-Associated Molecular Patterns (PAMPs) and heat-killed bacteria, and we created a database of reference spectra. The MALDI-TOF MS profiles of PBMCs from septic patients were then compared with the database. No differences were found between patients with documented infection (*n* = 6) and those without bacteriological documentation (*n* = 6). The spectra of PBMCs from septic patients matched with those of interferon-γ- and interleukin-10-stimulated PBMCs, confirming that sepsis is characterized by both inflammatory and immunoregulatory features. Interestingly, the spectra of PBMCs from septic patients without documented infection matched with the reference spectrum of PBMCs stimulated by CpG oligonucleotides, suggesting a bacterial etiology in these patients.

**Conclusions:**

Despite the limits of this preliminary study, these results indicate that the monitoring of functional status of PBMCs in peripheral blood by whole cell MALDI-TOF MS could provide unique opportunities to assess disease progression or resolution in clinical settings.

**Electronic supplementary material:**

The online version of this article (10.1186/s12879-018-3266-7) contains supplementary material, which is available to authorized users.

## Background

Sepsis is the combination of a systemic inflammatory response syndrome (SIRS) and infection [1]. Sepsis can progress to severe sepsis and septic shock, with mortality rates of 25 to 30% and 40 to 70%, respectively [2, 3]. The clinical manifestations of the early stages of sepsis are often similar to those of a patient with SIRS caused by sterile inflammation [3], leading to frequent underappreciation of sepsis in clinical practice. The traditional approach to sepsis diagnosis is based on the clinical signs and symptoms of sepsis, supported by relevant microbiological data. Unfortunately, up to 40% of the infections suspected in patients with sepsis are not microbiologically documented [4–6]. Consequently, physicians often use empiric antibiotic therapy, which has three major drawbacks: increased antibiotic resistance, patient toxicity, and elevated costs [7].

Two important challenges for physicians are to determine if the patient is infected or not in the absence of microbiological documentation, and when to begin antimicrobial therapy [8–10]. Numerous molecules, such as procalcitonin, are unable to discriminate between sepsis and SIRS [11, 12]. Cytokine imbalance has been thought to be useful for defining sepsis. Indeed, the recognition of soluble Pathogen-Associated Molecular Patterns (PAMPs) such as lipopolysaccharide (LPS), peptidoglycan (PGN), CpG oligonucleotides (CpG-ODN), and polyinosinic polycytidylic acid (poly I:C) by pathogen recognition receptors (PRRs) induces the release of inflammatory cytokines, such as gamma interferon (IFN-γ) and the concomitant liberation of immunoregulatory cytokines, including interleukin (IL)-10 and IL-4. However, cytokine profiles are also unable to discriminate sepsis, and seem more related to SIRS severity than to sepsis [13].

MALDI-TOF mass spectrometry (MS) has emerged as a fast, reliable and inexpensive tool for bacterial identification and diagnosis [14, 15]. Interestingly, bacterial identification does not require previous fractionation steps [16]. Recently, we and others have applied the whole-cell MALDI-TOF MS technique to identify eukaryotic cells, including circulating cells [17–19]. Our team has also shown that whole-cell MALDI-TOF MS detects the multifaceted activation of monocyte-derived macrophages in response to various cytokines and bacterial pathogens [20]. Portevin et al. [21] demonstrated recently that MALDI-TOF MS fingerprints distinguish human monocyte sub-populations activated by distinct microbial ligands.

Our goal was to analyze peripheral blood mononuclear cells (PBMCs) in septic patients through whole-cell MALDI-TOF MS. This approach enabled the detection of a specific PBMC signature in septic patients. The analysis of the signature of healthy PBMCs stimulated with cytokines, soluble PAMPs and bacteria, frequently involved in sepsis, showed that the spectra of PBMCs from septic patients matched with those of PBMCs stimulated by IFN-γ, IL-10 and CpG-ODN. These findings evoked an infectious activation in septic patients regardless of documented or undocumented infection. Despite the limits of this preliminary study, this is the first report describing the use of a whole-cell MALDI-TOF MS approach to identify PBMC activation in septic patients.

## Methods

### Ethics statement

The study was approved by the Ethics Committee of the Assistance Publique-Hôpitaux de Marseille, France. Blood was collected after informed and written consent of healthy donors and septic patients.

### Patients and healthy controls

Patient recruitment was provided from an ancillary study to the project “De-escalation of Empirical Antibiotics in Severe Sepsis” (Comité de Protection des Personnes Sud Méditerranée No. 2011–002297-22). Twelve patients (aged 18 and over) were enrolled in the polyvalent intensive care unit (North Hospital, Marseille, France). A single blood sample was collected at the time of empirical antibiotic initiation. Eligibility criteria were the presence of severe sepsis requiring empirical antimicrobial treatment. Severe sepsis was defined as the criteria for SIRS and suspected infection with at least one organ failure. SIRS was defined by two or more of the following conditions: temperature > 38 °C or < 36 °C, heart rate > 90 beats per minute, respiratory rate > 20 breaths per minute or PaCO2 < 32 mmHg, white blood cell count >12G/l, < 4G/l, or > 10% immature cells. Two patients with SIRS had *Staphylococcus aureus* bacteremia, 4 had gram-negative bacillus bacteremia and 6 had a strongly suspected infection clinically but not microbiologically documented. PBMCs from healthy donors were isolated from leukopacks (Etablissement Français du Sang).

### Isolation and in vitro activation of PBMCs

Blood was collected in EDTA-containing tubes and PBMCs were isolated using Ficoll cushions (MSL, Eurobio). After centrifugation, PBMCs were washed in sterile phosphate buffered saline (PBS) without Ca^2+^ and Mg^2+^, and 1 × 10^6^ cells were suspended in 10 μL of PBS and frozen at − 80 °C for 2 to 3 days before analysis. In some experiments, PBMCs from healthy donors (1 × 10^6^ cells in 6-well plates) were incubated in 2 mL of RPMI 1640 containing 10% fetal calf serum (FCS) and stimulated with 20 ng/mL IFN-γ (PeproTech), 20 ng/mL IL-4 or IL-10 (R&D Systems) for 18 h [22]. PBMCs were also stimulated with LPS from *Escherichia coli* (1 μg/mL, Sigma-Aldrich), peptidoglycan (PGN) from *Bacillus subtilis* (10 μg/mL, Sigma-Aldrich), CpG-ODN (2 μg/mL, InvivoGen) and poly I:C (25 μg/mL, InvivoGen). Finally, PBMCs were stimulated with heat-inactivated bacteria (10 bacteria per cell). Bacteria included oxacillin-sensitive *S. aureus*, community strain group B streptococcus, *Pseudomonas aeruginosa* (ATCC 27853) and *E. coli* (ATCC 25922). PBMCs stimulated for 18 h were pelleted in 10 μl of PBS and frozen as unstimulated PBMCs for MALDI-TOF MS analysis.

### MALDI-TOF MS analysis

After thawing of the PBMCs, 1 μL of cell suspension was added to 1 μL of matrix solution (saturated solution of α-cyano-4-hydroxy-cynnamic acid in a mixture of 50% acetonitrile, 25% trifluoroacetic acid and water) as previously described [17, 20, 23]. The mixture was deposited on the MALDI target. The evaporation that gradually took place at room temperature allowed the formation of α-cyano-4-hydroxy-cynnamic acid crystals containing the dispersed samples. Measurements were performed using an Autoflex II mass spectrometer (Bruker Daltonics, Wissembourg, France) equipped with a 337-nm nitrogen laser. Each sample was irradiated with a laser for desorption and ionization. Each spectrum resulted from the sum of positive ions obtained after 525 laser shots in different regions of the analyzed spot (automatic mode). All the positive-ion mass spectra were acquired in the linear mode at an acceleration voltage of 20 kV in the delayed extraction mode. A signal-to-noise of 3.0 was selected to define peaks, with a maximum of 100 peaks per spectrum. Spectra were automatically acquired with a mass/charge (*m/z*) ranging from 2000 to 20,000 Da using FlexControl and FlexAnalysis 2.4 software (Bruker Daltonics). The x-axis of spectra represented the *m/z* ratio (in daltons) of ionized molecules, and the y-axis indicated the intensity (relative abundance) of these ions.

### Spectrum analysis

Analyses and graphical outputs were performed using the free and open source statistical analysis software R (version 2.13), along with specific analysis libraries (MALDIquant) as previously described [20]. The gel view representation indicates the reproducibility of the spectra. A hierarchical clustering with a ward algorithm for agglomeration and a dissimilarity matrix based on the Jaccard distance were used to classify the spectra. The MALDI Biotyper 3 software (Bruker Daltonics) was used to create an average reference spectrum for each PBMC sample, corresponding to at least 10 individual spectra. The Biotyper software realigns acquired spectra and automatically creates an average spectrum using default Biotyper software settings provided by the manufacturer, and we created a database as previously described [20]. The Biotyper software also allows the identification of unknown spectra as shown in clinical samples by comparison with reference spectra, for the identification and classification of microorganisms [14]. The score values proposed by the manufacturer have been used for microorganism identification. The score values between 0.000 and 1.699 do not allow reliable microbe identification; the values between 1.700 and 1.999 allow probable cell identification and score values higher than 2.0 are considered statistically significant; they allow the confident identification of different microbe species. We extended this method to assess PBMC activation status in septic patients. As the score values provided by Bruker Daltonics ranged from 0.000 to 2.000 when we used control samples and reference spectra, we considered that medians of matching scores higher than 1.5 allowed reliable identification of the activation profile of patient PBMCs and could be clinically relevant. Differences between conditions were tested with the Mann-Whitney *U* test and a cutoff value of 0.05 was chosen to consider a difference statistically significant.

## Results

### Signatures of PBMCs

**Fig. 1:**
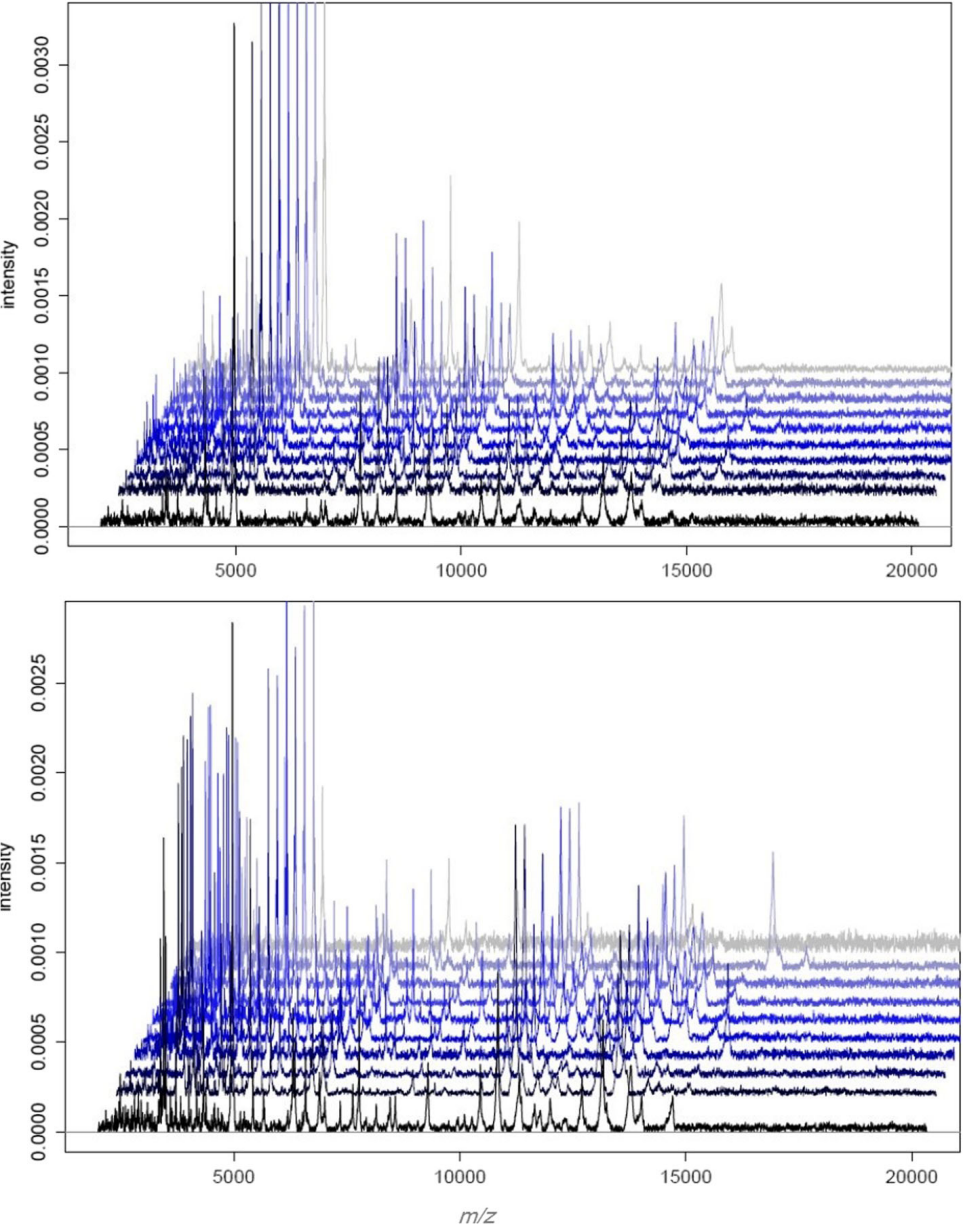
MALDI-TOF MS spectra of PBMCs. The PBMCs (1 × 10^6^ cells) from ten healthy donors (**a**) and ten septic patients (**b**) were suspended in 10 μL of PBS, and 1 μL was deposited onto the MALDI target. Representative MALDI-TOF MS spectra are shown. MALDI-TOF MS spectra were analyzed using statistical analysis software R (version 2.13)

**Fig. 2:**
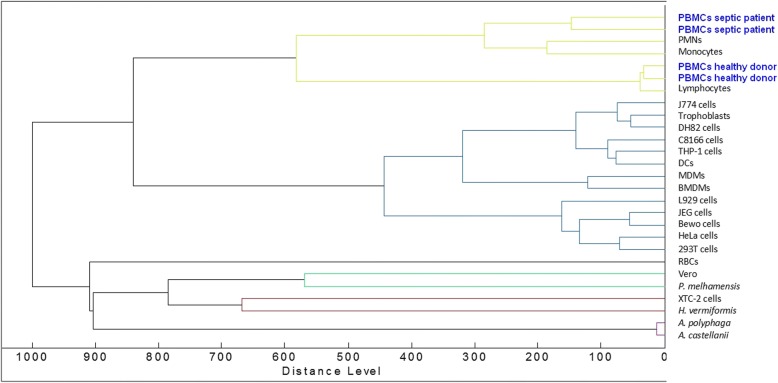
Dendrogram representation of PBMCs**.** The dendrogram constructed by Ouedraogo et al. [17] was implemented with reference spectra of PBMCs from two healthy donors and two septic patients. The Biotyper (Bruker Daltonics) software was used to create an averaged spectrum for each patient, corresponding to at least 10 individual spectra. The averaged spectra were added to the database using the Biotyper software and the dendrogram creation method. Peripheral blood mononuclear cells (PBMCs); polymorphonuclear cells (PMNs); dendritic cells (DCs); monocyte-derived macrophages (MDMs); bone marrow-derived macrophages (BMDMs); red blood cells (RBCs)

As compared to phenotype studies that require panels of antibodies to characterize circulating cells, MALDI-TOF MS enables simple and rapid measurement of cell status. First, we wondered if PBMCs from healthy controls were characterized by a specific whole-cell MALDI-TOF MS signature. The spectra of PBMCs from ten healthy donors were composed of numerous peaks ranging from 2000 to 15,000 Da, with a major peak at 4961 Da. Note that the spectra of the ten donors were highly reproducible (Fig. 1a). Second, we found that the signatures of PBMCs from ten septic patients were similar but they were distinct from those of PBMCs from healthy controls. Because MALDI-TOF MS profiles were specific for PBMCs from healthy controls and septic patients, the dendrogram constructed by Ouedraogo et al. [17] was implemented with reference spectra of PBMCs from two healthy donors and two septic patients. As expected, the PBMCs from the two healthy donors were similar and clustered with T lymphocytes and, to a lesser extent, with monocytes and polymorphonuclear cells (PMNs). Likewise, the PBMCs from the two septic patients were similar and formed a specific cluster. This cluster was close to that of monocytes and PMNs but was away from the T lymphocyte cluster (Fig. 2). This specific pattern underlines the prominent role of the innate immune response in sepsis. Taken together, these results demonstrated that PBMC fingerprints distinguished patients with sepsis from healthy controls.

### Peak characteristics of PBMCs in sepsis

We wondered if the analysis of individual peaks enables stratification of patients with sepsis, microbiologically documented or not. When the spectra were analyzed by comparing the presence or absence of peaks, we found that 10 peaks were present only in controls, 12 peaks were common to healthy donors and septic patients and 18 peaks were specific in septic patients (Table 1). Taking into account the limitations related to the limited number of patients in this preliminary study, among these 18 specific peaks, 15 were common to all septic patients independently of microbiological documentation. Concerning the three other peaks, the peak at 5415 Da was found in patients with gram-negative bacillus bacteremia (4/4) and one patient without microbiological documentation. The peak at 3329 Da was present in 3/4 patients with gram-negative bacillus bacteremia and the same patient without bacteriological documentation, suggesting that these two peaks could be associated with gram-negative bacillus bacteremia and that this patient without microbiological documentation could have gram-negative bacillus sepsis. Finally, the peak at 2942 Da was found in the two patients with *S. aureus* bacteremia and in one patient without microbiological documentation, suggesting that this peak could be related to *S. aureus* bacteremia (Table 1). Despite the limits of the study, these results show that the signature of septic patients is very similar, regardless of documented or undocumented infection.

**Table 1:** Peak characteristics of PBMCs

Healthy donors	Gram-negative bacillus bacteremia	*S. aureus* bacteremia	Patients without documented infection
2165			
2227			
2302			
2503			
	2617	2617	2617
	2631	2631	2631
	2646	2646	2646
2777			
	2795	2795	2795
		2942	2942
	3329		3329
	3355	3355	3355
3363			
3369	3369	3369	3369
	3398	3398	3398
	3426	3426	3426
3441	3441	3441	3441
3455			
	3467	3467	3467
3485	3485	3485	3485
	3708	3708	3708
3881			
	3998	3998	3998
	4323	4323	4323
4642			
4935	4935	4935	4935
4961	4961	4961	4961
4983	4983	4983	4983
5023			
	5415		5415
	6298	6298	6298
	6342	6342	6342
6574	6574	6574	6574
	7621	7621	7621
7762	7762	7762	7762
8561	8561	8561	8561
9285	9285	9285	9285
10,259	10,259	10,259	10,259
	10,441	10,441	10,441
10,831	10,831	10,831	10,831

The PBMCs from healthy donors and septic patients were analyzed by MALDI-TOF MS

The *m/z* ratio of peaks is shown. The peaks that were common to septic patients are underlined

### Signatures of PBMCs activated in vitro

**Fig. 3:**
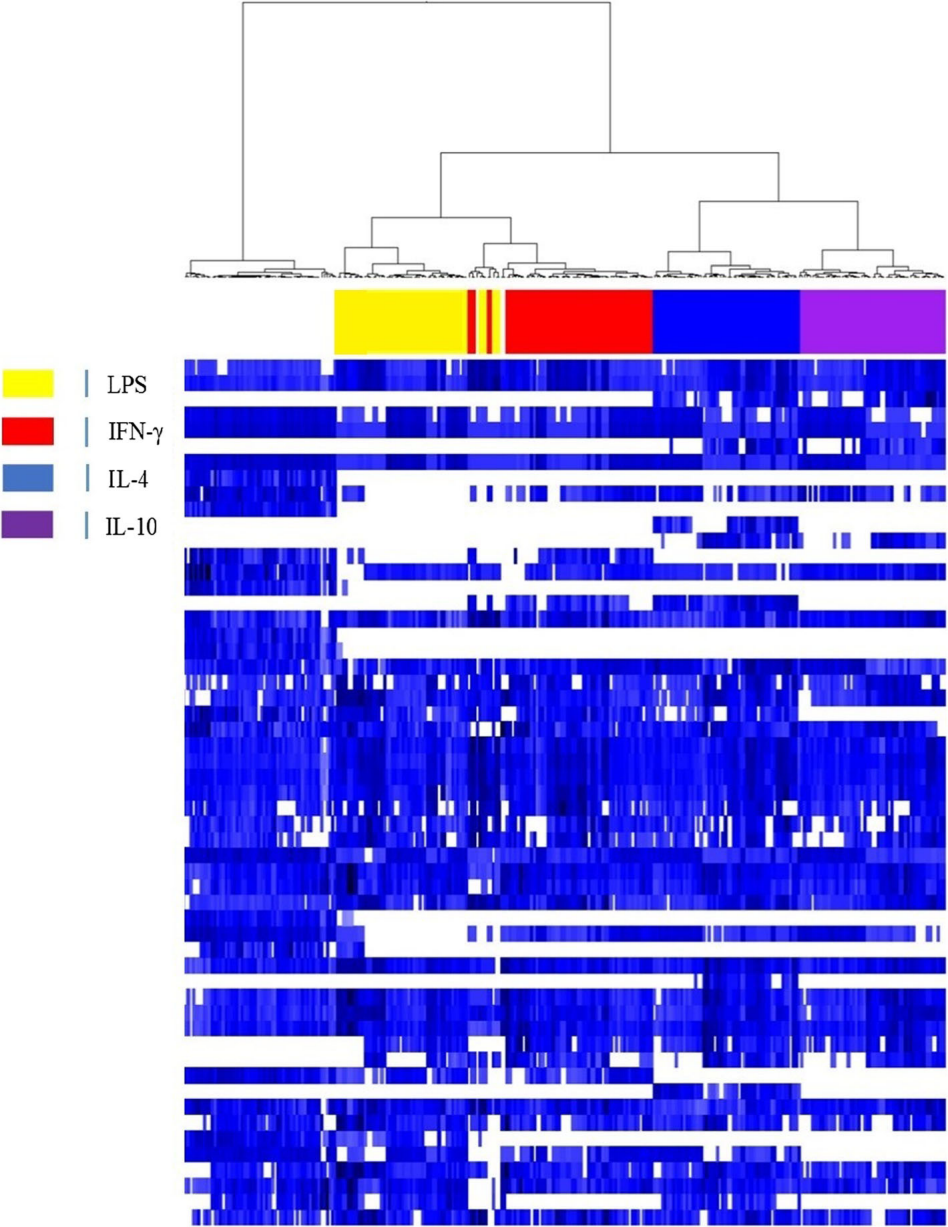
Gel view representation of activated PBMCs. The PBMCs from healthy donors were stimulated with 20 ng/ml IFN-γ, IL-4, IL-10 or 1 μg/mL LPS for 18 h. The spectra were arranged in a pseudo-gel format using a gel view representation. Vertical axis refers to the m/z ratio. Spectra are classified according to the presence/absence of peaks. Unstimulated PBMCs are presented in white

Because the differences between the signatures of healthy and septic PBMCs may be related to their activation state, we stimulated PBMCs with various agonists to determine if PBMC activation results in specific profiles. The stimulation of PBMCs with IFN-γ, LPS, IL-4 and IL-10 led to specific and reproducible responses, as shown using a virtual gel view representation (Fig. 3). The spectra from all stimulated samples were clearly separated from those of unstimulated PBMCs. Each type of stimulation led to specific fingerprints. The spectra from the PBMCs stimulated with inflammatory molecules (IFN-γ/LPS) clustered together. Similarly, immunoregulatory cytokines induced the clustering of PBMCs. The existence of these three major clusters (unstimulated, IFN-γ/LPS-, IL-4/IL-10-stimulated PBMCs) suggests that MALDI-TOF MS may be used to analyze the inflammatory response of PBMCs in the clinical setting.

**Fig. 4:**
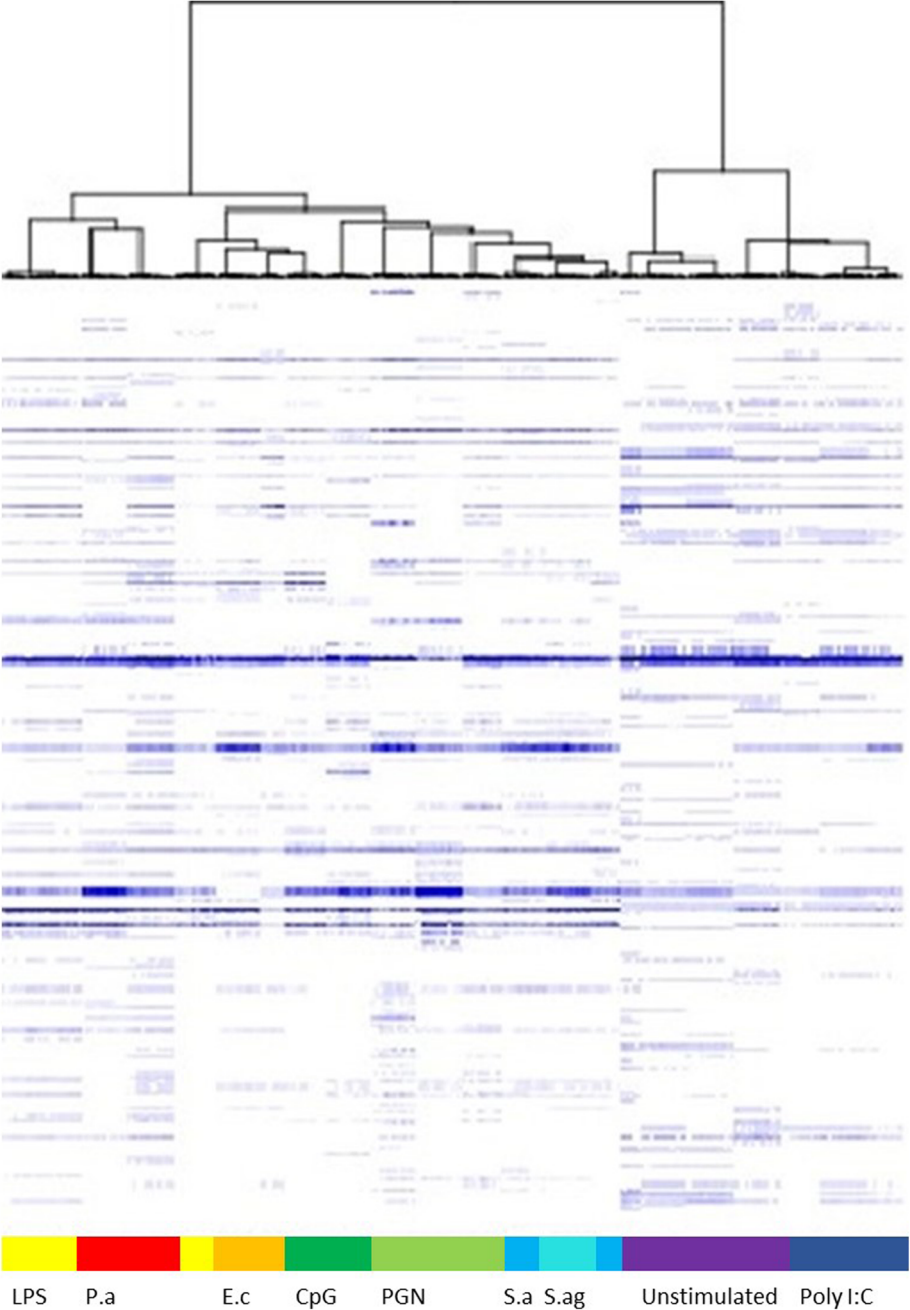
Hierarchical clustering of activated PBMCs. PBMCs were stimulated with different agonists for 18 h. The results are shown as hierarchical clustering. Vertical axis refers to the m/z ratio. Spectra are classified according to the presence/absence of peaks. PBMCs stimulated with LPS from *Escherichia coli* are presented in yellow, with *Pseudomonas aeruginosa* (*P.a*) in red, *Escherichia coli* (*E.c*) in orange, CpG ODN in dark green, PGN in green, *Staphylococcus aureus* (*S.a*) in blue, *Streptococcus agalactiae* (*S.ag*) in turquoise, unstimulated PBMCs in purple and PBMCs stimulated with poly I:C in dark blue

We also investigated the responses of PBMCs to PAMPs and bacteria often found in sepsis. For that purpose, we used unstimulated PBMCs as controls and we compared the fingerprints induced by PAMPs and bacteria to these controls. Two very different clusters were clearly identified: one including unstimulated and poly I:C-stimulated PBMCs and the other with PBMCs stimulated with bacteria and other PAMPs (Fig. 4). Among the bacterial signatures, we observed that the signatures induced by *P. aeruginosa* and *E. coli* (Gram negative bacteria) were coupled to the signature induced by LPS from *E. coli.* Similarly, the signatures induced by *S. agalactiae* group B and *S. aureus* (gram-positive bacteria) were associated and close to the signature induced by PGN from *B. subtilis*. These results suggest that MALDI-TOF MS may be useful to assess a series of specific responses of PBMCs to varied agonists.

**Fig. 5:**
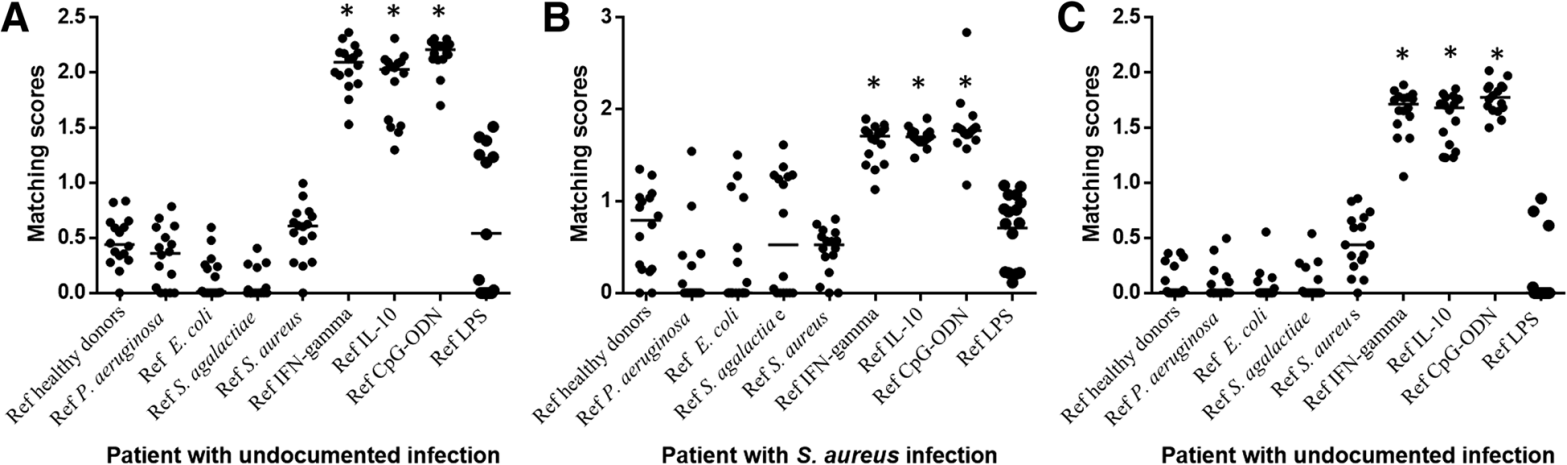
Comparison between in vitro and in vivo data. Averaged spectra of PBMCs stimulated in vitro by different agonists were generated from the database using the Biotyper software. The spectra (*n* = 16) from four patients with *E. coli* bacteremia, two patients with *S. aureus* bacteremia and six patients with undocumented infection were then compared with the averaged spectra of the database. Scatter plots of one representative septic patient with *E. coli* infection (**a**), *S. aureus* infection (**b**) or without microbiological documentation (**c**) are presented. Matching scores between each spectrum from patients and averaged spectra from the database are represented with circles. Horizontal lines represent the medians of matching scores; a value higher than 1.5 was considered significant and allowed confident identification of the activation status of PBMCs. The nonparametric Mann-Whitney *U* test was used to compare scores with the averaged spectra of the database

Finally, we used matching scores to compare the different modes of PBMC activation after having created averaged spectra of stimulated PBMCs. The MALDI-TOF MS profiles of PBMCs from septic patients were then compared with the database. None of the septic patients matched with the averaged spectrum of healthy donors, while the spectra of septic patients significantly matched with IFN-γ and IL-10 spectra regardless of whether the infection was documented (*n* = 6) or not (n = 6), confirming that sepsis is characterized by both inflammatory and immunoregulatory features (Fig. 5 and Additional file 1: Figure S1). It is noteworthy that the scores obtained by comparing the spectra of *E. coli*- and *S. aureus*-infected patients with the reference spectrum of *E. coli*- and *S. aureus*-stimulated PBMCs were significantly lower (*p* < 0.05) than those obtained with IL-10- and IFN-γ-specific spectra, respectively (see Fig. 5a, b), suggesting the prominence of the activation profile as a specific response to pathogens. Importantly, we found that the spectra of PBMCs from septic patients significantly (*p* < 0.05) matched with CpG-ODN, independently of a documented infection. Clearly, the activation profile found in the patients with unknown infection was similar to that of the patients with documented infection (see Fig. 5c), suggesting that these patients had a bacterial infection. Taken together, these results are consistent with that septic PBMCs were activated in a specific way and suggest a bacterial infection in septic patients without documented infection.

## Discussion

Sepsis is a frequent and serious complication in intensive care unit patients. Despite many years of active research to find effective and specific therapies, the only true treatment still relies on organ system support and effective antimicrobial eradication with antibiotics and/or surgical intervention. An important factor in optimizing survival rates in these patients is the speed of diagnosis [2–5]. However, diagnosing sepsis is not always straightforward, particularly in patients who have complex ongoing disease processes. In addition, many of these patients received antimicrobial therapy that rendered microbial cultures negative. Hence, 30–40% of intensive care unit patients with severe sepsis had negative bacterial cultures [4–6]. Even when cultures are positive, results take several days to become available, thus slowing the diagnostic process. Many biomarkers have been proposed over the years, but the diagnostic value of these molecules remains uncertain [24, 25].

We reasoned that a more integrated approach such as MALDI-TOF MS may be used to detect sepsis specifically in patients without microbiological documentation. As expected, we found that the signature of PBMCs from different healthy subjects was highly reproducible. It clustered with T lymphocytes but was largely different from the signature of numerous non-circulating cells. The signatures of PBMCs from septic patients largely differed from that of PBMCs from healthy controls. Interestingly, they clustered with the signature of monocytes and PMNs but not with T lymphocytes. The absence of clustering with T lymphocytes may be related to the lymphopenia associated with systemic inflammation syndromes [26]. This specific pattern underlines the prominent role of the innate immune response in sepsis.

We postulated that the fingerprints of septic patients may be related to specific activation of PBMCs. To assess the activation of PBMCs, we stimulated PBMCs from healthy controls with cytokines, PAMPs and bacteria. First, we identified both inflammatory (IFN-γ and LPS) and immunoregulatory (IL-4 or IL-10) signatures in PBMCs. Second, we also found that gram-negative bacteria and LPS induced specific signatures compared to those induced by gram-positive bacteria and PGN. Interestingly, the signatures induced by bacterial PAMPs were separate from a poly I:C, a PAMP known to strongly stimulate type 1 interferon as do most of viruses. This result might be useful in discriminating bacterial and viral infections, such as in pneumonia, for which no clinical, radiological and laboratory data differentiate bacterial from viral pneumonia [27].

Finally, we attempted to relate in vitro data and the fingerprints of septic PBMCs. We clearly identified IFN-γ and IL-10 signatures in septic patients. This result is consistent with the natural history of sepsis, where both inflammatory and immunoregulatory responses are observed [28]. We did not detect the signals delivered in vitro by heat-inactivated bacteria and bacterial ligands such as LPS and PGN, even when microbiological infection was documented. We can hypothesize that the lack of LPS and PGN signatures in sepsis may be related to anergy (endotoxin tolerance) [29]. In contrast, we found an intense CpG-ODN signature in septic patients, even in patients without microbiological documentation, suggesting the prominence of the activation profile as a specific response to pathogens. The Biotyper score cutoffs of 1.5 can be considered as unacceptably low for functions such as microbial identification. However, no comparison is possible because no score has so far been validated to discriminate specific responses of PBMCs to varied agonists. This is the first report describing the use of a whole-cell MALDI-TOF MS approach to identify PBMC activation in septic patients. As the score values provided by Bruker Daltonics ranged from 0.000 to 2.000 when we used control samples and reference spectra, we considered that medians of matching scores higher than 1.5 allowed reliable identification of the activation profile of patient PBMCs. Despite the choice of this score cutoff, we did not detect the signals delivered in vitro by heat-inactivated bacteria or bacterial ligands such as LPS and PGN in septic patients even with documented microbiological infection. Obviously, we observed a distinct and reproductible IFN-γ, IL-10 and CpG-ODN. The preliminary nature of the findings requires nevertheless confirmation of results.

To our knowledge, this is the first report describing the use of whole-cell MALDI-TOF MS analysis to identify mass spectra that discriminate specific responses of PBMCs to varied agonists and to study functional and activation status of septic patients with and without documented bacterial infection.

## Conclusions

This study shows that MALDI-TOF MS of patient PBMCs is easy and fast to perform and may be considered as a routine method for the detection of sepsis. The reproducibility and accuracy of this approach enables the analysis of several types of PBMC activation and shows a similar activation signature for septic patients with and without documented bacterial infection. Consequently, this innovative approach may be promising in helping physicians in the identification and prognosis of septic patients and/or their treatment. This proof of concept could easily be translated to clinical studies to monitor the functional status of PBMCs from patients under treatment and to study the activation status of PBMCs from patients suffering from systemic and chronic inflammatory disorders. However, the preliminary nature of the findings requires confirmation of results. As a next step, larger studies would confirm whether this new technique can improve the medical management of patients. High throughput monitoring of functional status of PBMCs in peripheral blood based on whole cell MALDI-TOF MS could provide unique opportunities to monitor disease progression and resolution in clinical settings.

## Additional file

Additional file 1: **Figure S1.** Comparison between in vitro and in vivo data. Averaged spectra of PBMCs stimulated in vitro by different agonists were generated from the database using the Biotyper software. The spectra (*n* = 16) from 2 other patients with gram-negative bacillus bacteremia, the second patients with *S. aureus* bacteremia and three other patients with undocumented infection were then compared with the averaged spectra of the database. Matching scores between each spectrum from patients and averaged spectra from the database are represented with circles. Horizontal lines represent the medians of matching scores; a value higher than 1.5 was considered significant and allowed confident identification of the activation status of PBMCs. (JPG 320 kb)

## Abbreviations

CpG-ODN: CpG oligonucleotides
*E. coli*: *Escherichia coli*
IFN-γ: Gamma interferon
IL: Interleukin
LPS: Lipopolysaccharide
MS: Mass spectrometry
PBMCs: Peripheral blood mononuclear cells
PGN: Peptidoglycan
Poly I:C: Polyinosinic polycytidylic acid
PRRs: Pathogen recognition receptors
*S. aureus*: *Staphylococcus aureus*
SIRS: Systemic inflammatory response syndrome
