# Supplementary figures and images for: MALDI-TOF MS monitoring of PBMC activation status in sepsis
Source: BMC Infect Dis. 2018 Jul 31;18:355. doi: 10.1186/s12879-018-3266-7 (PMC6069833; doi:10.1186/s12879-018-3266-7)

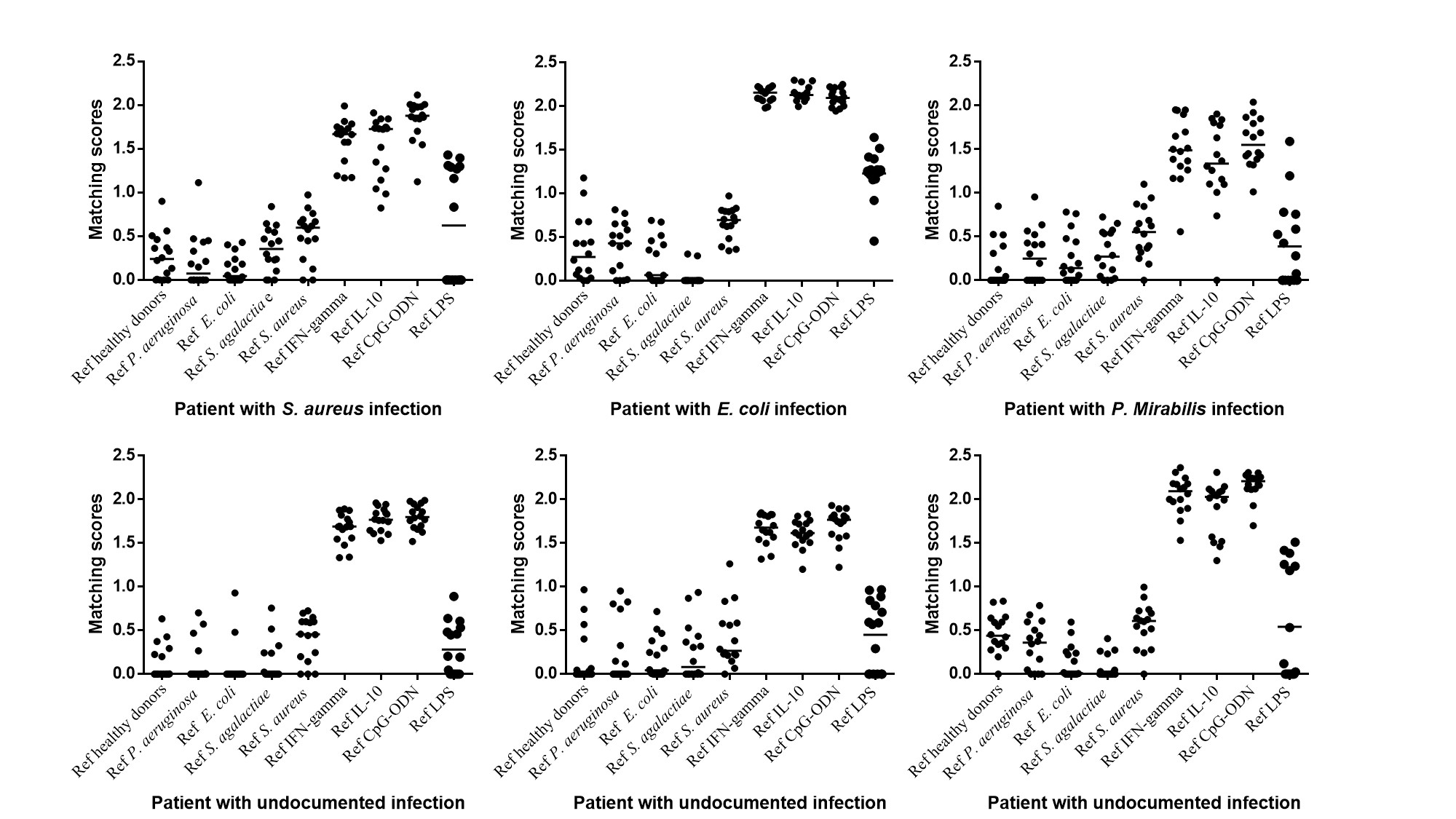

Supplement: Supplementary file 1 — Figure S1. Comparison between in vitro and in vivo data. Averaged spectra of PBMCs stimulated in vitro by different agonists were generated from the database using the Biotyper software. The spectra (n = 16) from 2 other patients with gram-negative bacillus bacteremia, the second patients with S. aureus bacteremia and three other patients with undocumented infection were then compared with the averaged spectra of the database. Matching scores between each spectrum from patients and averaged spectra from the database are represented with circles. Horizontal lines represent the medians of matching scores; a value higher than 1.5 was considered significant and allowed confident identification of the activation status of PBMCs. (JPG 320 kb) [file 12879_2018_3266_MOESM1_ESM.jpg]
